# Telmisartan Lowers Elevated Blood Pressure in Psoriatic Mice without Attenuating Vascular Dysfunction and Inflammation

**DOI:** 10.3390/ijms20174261

**Published:** 2019-08-30

**Authors:** Johannes Wild, Rebecca Schüler, Tanja Knopp, Michael Molitor, Sabine Kossmann, Thomas Münzel, Andreas Daiber, Ari Waisman, Philip Wenzel, Susanne Helena Karbach

**Affiliations:** 1 Center for Cardiology—Cardiology I, University Medical Center Mainz, 55131 Mainz, Germany; 2 Center for Thrombosis and Hemostasis (CTH), University Medical Center Mainz, 55131 Mainz, Germany; 3 German Center for Cardiovascular Research (DZHK)—Partner site Rhine-Main, 60590 Frankfurt, Germany; 4 Institute of Molecular Medicine, University Medical Center Mainz, 55131 Mainz, Germany; 5 Heart Research Institute, 2042 Sydney, Australia

**Keywords:** psoriasis, vascular dysfunction and inflammation, Interleukin-17A, hypertension and anti-hypertensive treatment, telmisartan

## Abstract

Background: Psoriasis is hallmarked by vascular dysfunction, arterial hypertension, and an increased risk for cardiovascular diseases. We have shown recently that skin-driven interleukin (IL)-17A expression promotes psoriasis-like disease in mice, and this is associated with vascular inflammation, vascular dysfunction, and hypertension. As an intensive risk-factor reduction is recommended for psoriasis patients, we aimed to elucidate the impact of the angiotensin II receptor type 1 (AT1) antagonist telmisartan in a mouse model of severe psoriasis-like skin disease. Methods and Results: Elevated blood pressure measured by tail-cuff plethysmography in mice with keratinocyte-specific IL-17A overexpression (K14-IL-17A^ind/+^ mice) was significantly reduced in response to telmisartan. Importantly, vascular dysfunction, as assessed by isometric tension studies of isolated aortic rings, vascular inflammation measured by flow cytometry analysis of CD45^+^CD11b^+^ immune cells, as well as the increased peripheral oxidative stress levels assessed by L-012-enhanced chemiluminescence were not attenuated by telmisartan treatment of K14-IL-17A^ind/+^ mice, nor was the persisting skin inflammation. Conclusion: We provide first evidence for an effective antihypertensive treatment in experimental psoriasis by AT1 blockade, but without any impact on vascular inflammation and dysfunction in our mouse model of severe psoriasis-like skin disease. This suggests that vascular function and inflammation in psoriasis might not be attenuated as long as skin inflammation persists.

## 1. Introduction

Patients with severe psoriasis are at increased risk for cardiovascular comorbidities [1,2,3]. Over a long period, the behavioral-driven risk factors in psoriasis patients were assumed to be responsible for this association. Currently, we know that the inflammatory components of the interleukin (IL)-17A-driven skin disease play a striking role in linking severe psoriasis and cardiovascular mortality. In clinical practice, however, there is still a lack of awareness of the link between skin and cardiovascular disease, resulting in an enduring under-treatment of cardiovascular risk factors in psoriasis patients [4,5]. The Framingham Heart Study outlined the classical modifiable cardiovascular risk factors: smoking, diabetes, physical inactivity, obesity, dyslipidemia, and arterial hypertension [6]. Of these, hypertension remains the major preventable cause of cardiovascular disease and all-cause mortality worldwide [7]. Hypertension is more prevalent among patients with psoriasis [8,9] and the likelihood of poorly controlled hypertension appears to increase with more severe skin disease [10]. Considering these aspects, an effective antihypertensive treatment is of fundamental importance to reduce cardiovascular mortality and morbidity in psoriasis. Surprisingly, the cardiovascular effects of commonly used antihypertensive drugs have not been investigated in the special population of psoriasis patients up to now.

We have shown before that dermal over-expression of the cytokine IL-17A (K14-IL-17A^ind/+^) in mice results in severe psoriasis-like skin disease and simultaneous endothelial dysfunction, vascular inflammation, and arterial hypertension [11]. Thus, this mouse strain mimics the cardiovascular comorbidity of patients with severe psoriasis, which makes it a convenient model for analyzing cardiovascular treatment options in severe psoriasis. Anti-inflammatory therapy with anti-IL-17A attenuated peripheral oxidative stress levels in this model of severe psoriasis and was highly efficient in our mouse model of moderate to severe psoriasis in reducing vascular inflammation and inflammation as well as oxidative stress levels and skin inflammation itself [12].

As patients with severe psoriasis are high-risk patients from the cardiovascular point of view [2], an intensive risk-factor reduction is recommended [13]. Thus, we asked ourselves if common cardiovascular medication to lower high blood pressure reduces hypertension as well as vascular dysfunction and inflammation in our mouse model of severe psoriasis. As drugs that affect the renin-angiotensin-aldosterone system (RAAS) are the most widely used class of antihypertensive drugs [7] that also include an anti-inflammatory capacity, we focused on the angiotensin II receptor blocker (ARB) telmisartan. This active substance is routinely used in human patients and has been studied in murine organisms before [14]. So, the goal of our study was to elucidate the impact of oral telmisartan treatment in our mouse model of severe psoriasis-like skin disease with typical cardiovascular comorbidities.

## 2. Results

**Figure 1:**
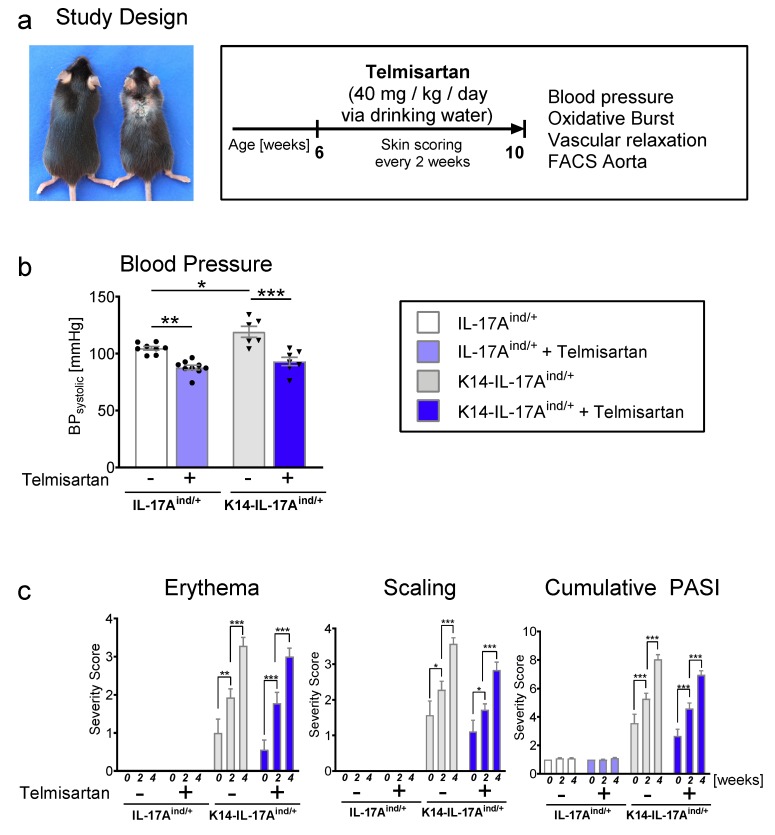
Telmisartan treatment lowers blood pressure in mice with severe psoriasis-like skin disease with no impact on skin disease. (**a**) Representative picture of a healthy control mouse and a K14-IL-17A^ind/+^ mouse (first published by Croxford et al. [15]) and description of the experimental design of our study. (**b**) Systolic blood pressure (BP) recordings by tail-cuff after four weeks of telmisartan treatment in K14-IL-17A^ind/+^ and control mice, n = 6–9, one-way ANOVA with Bonferroni’s post-test. (**c**) Erythema and scaling were scored in K14-IL-17A^ind/+^ and control mice with and without telmisartan treatment. Cumulative Psoriasis Area and Severity Index (PASI) score calculated. n = 6–9, two-way analysis of variance with Bonferroni multiple comparisons test. The p-values of ≤0.001, ≤0.01, and ≤0.05 were considered statistically significant and marked by three, two, and one asterisks, respectively, ns = not significant. All data are presented as mean ± SEM.

K14-IL-17A^ind/+^ and IL-17A^ind/+^ control mice were treated with high dose telmisartan (40 mg/kg bodyweight/day) via drinking water over a period of four weeks. Due to the progressive development of skin disease and vascular dysfunction with proceeding age in this mouse model, we decided to start the treatment at the age of six weeks and to use a high-dose regime (Figure 1a). After four weeks of telmisartan treatment, we found a highly significant decrease of systolic blood pressure in K14-IL-17A^ind/+^ and IL-17A^ind/+^ control mice compared to sham-treated littermates (Figure 1b). Telmisartan-treated K14-IL-17A^ind/+^ psoriasis mice even showed a similar systolic blood pressure as telmisartan-treated control mice. This finding indicates that the blood pressure lowering effects of the ARB telmisartan is also efficient for the hypertension occurring in psoriasis-like skin disease. The severity of skin disease was assessed every second week by quantification of erythema, scaling, skin thickness, the affected area, and cumulative Psoriasis Area and Severity Index (PASI). We found an increase of all skin parameters and combined PASI score in treated and untreated K14-IL-17A^ind/+^ mice during the four weeks of treatment, reflecting the increasing severity of psoriasis-like skin disease in psoriatic mice with proceeding age. As expected, the skin phenotype of the K14-IL-17A^ind/+^ mice was not at all altered by telmisartan treatment (Figure 1c). Of note, telmisartan treatment had increased mortality in K14-IL-17A^ind/+^ mice as a side effect (Supplementary Figure S1a).

**Figure 2:**
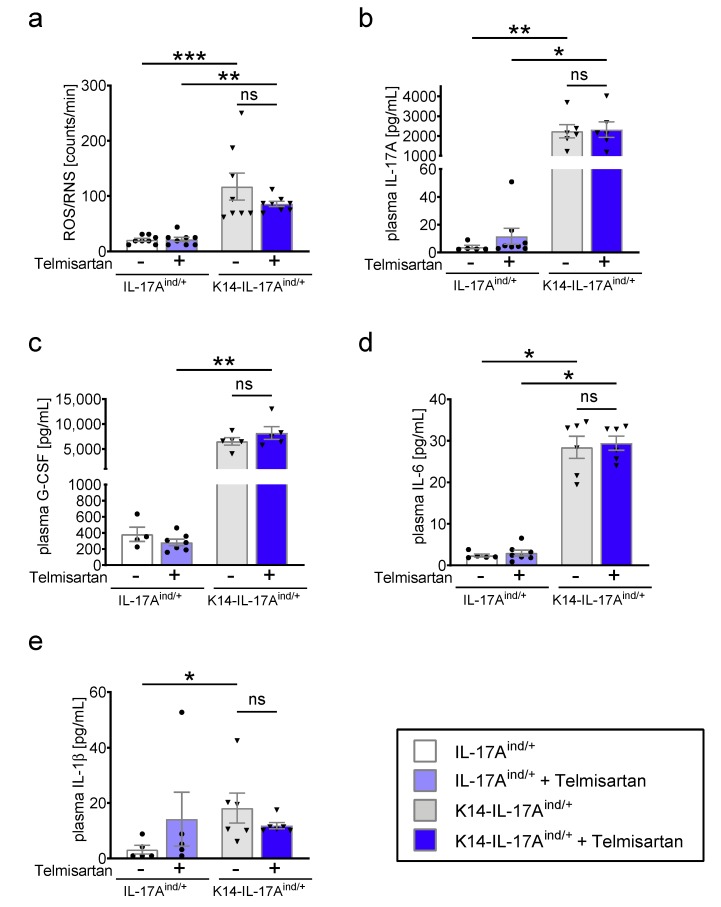
Persistently increased peripheral oxidative stress levels in telmisartan-treated mice suffering from severe psoriasis. (**a**) Reactive oxygen and nitric species (ROS/RNS) measurement of whole blood of K14-IL-17A^ind/+^ and control mice with and without telmisartan treatment. n = 8, one-way ANOVA with Bonferroni’s post-test. (**b**–e) Luminex measurement for b: interleukin (IL)-17A, (**c**): granulocyte-colony stimulating factor (G-CSF), (**d**): IL-6, and (**e**): IL-1β in plasma of K14-IL-17A^ind/+^ and control mice with and without telmisartan treatment. n = 5–8, Kruskal–Wallis one-way ANOVA with Dunn’s multiple comparisons test. IL-17A^ind/+^ mice are presented with black circles and K14-IL-17A^ind/+^ mice are presented in black triangles. The p-values of ≤0.001, ≤0.01, and ≤0.05 were considered statistically significant and marked by three, two, and one asterisks, respectively, ns = not significant. All data are presented as mean ± SEM.

After four weeks of treatment, we performed measurements of the oxidative burst in whole blood and aortic relaxation studies. We found no effect of telmisartan treatment on the significantly elevated superoxide levels (reactive oxygen and nitric species, ROS/RNS) in the blood of K14-IL-17A^ind/+^ mice (Figure 2a). In parallel, the increased cytokine levels of IL-17A, granulocyte-colony stimulating factor (G-CSF), IL-6, and IL-1β in the plasma of K14-IL-17A^ind/+^ mice were not lowered under telmisartan (Figure 2b–e), providing a stimulating and activating effect on myeloid cells, which are one important source of oxidative stress formation in chronic inflammation. The other cytokines analyzed in the plasma of K14-IL-17A^ind/+^ mice had no relevant increase compared to control mice, stressing the mainly IL-17A-driven myeloid activation cascade in psoriasis (Supplementary Figure S1b).

**Figure 3:**
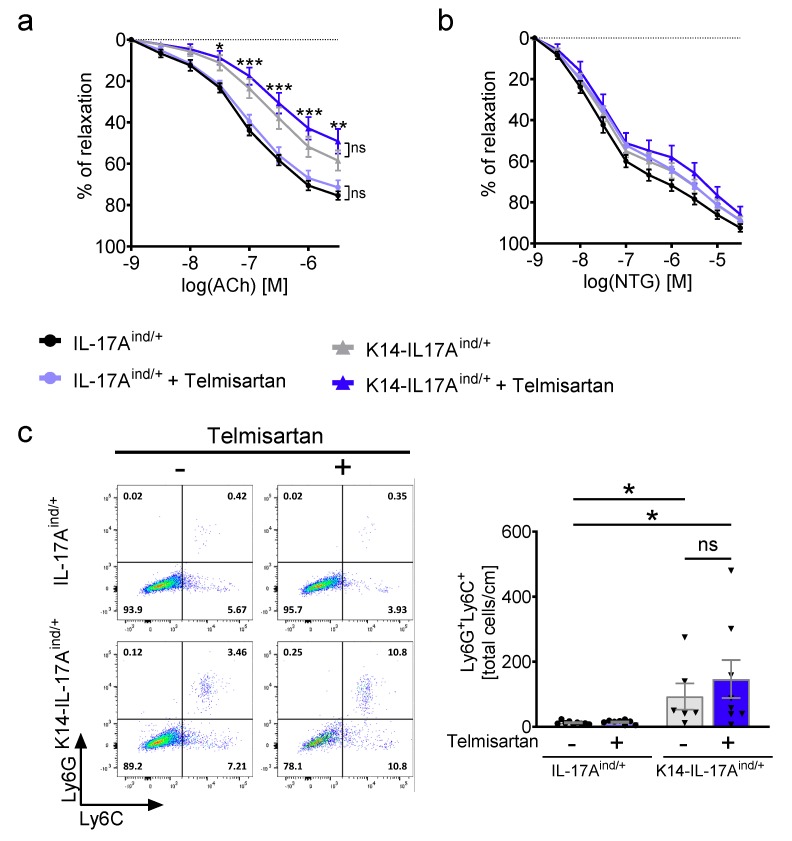
Telmisartan treatment does not attenuate vascular dysfunction and vascular inflammation in mice with severe psoriasis. (**a**) Isometric tension studies of aortic rings in response to Acetylcholine (Ach) for endothelium-dependent vascular relaxation. Two-way analysis of variance test and Bonferroni post hoc test, n = 6–9. Asterisks indicate significances between K14-IL-17A^ind/+^ and IL-17A^ind/+^ control mice. (**b**) Isometric tension studies of aortic rings in response to nitroglycerine (NTG) for endothelium independent vascular relaxation. Two-way analysis of variance test and Bonferroni post hoc test, n = 6–9. (**c**) Flow-cytometric analysis of aortas. Representative plots are shown for each group. The total cell number of Ly6G^+^Ly6C^+^ neutrophils per 1 cm of aorta is presented. Cells were pre-gated on living CD45.2^+^ and CD11b^+^ cells, n = 6–9. Kruskal–Wallis one-way ANOVA with Dunn multiple comparisons tests. IL-17A^ind/+^ mice are presented with black circles and K14-IL-17A^ind/+^ mice are presented in black triangles. The p-values of ≤0.001, ≤0.01, and ≤0.05 were considered statistically significant and marked by three, two, and one asterisks, respectively, ns= not significant. All data are presented as mean ± SEM.

In line with our previous data and with the increased oxidative stress levels in the blood periphery, aortas isolated from K14-IL-17A^ind/+^ animals revealed a severe endothelium-dependent vascular dysfunction at baseline (aortic relaxation in response to acetylcholine; Figure 3a), whereas smooth muscle-cell dependent relaxation was not altered (aortic relaxation in response to nitroglycerine; Figure 3b) [11,12]. Despite the normalized blood pressure, treatment with high-dose telmisartan did not improve the vascular relaxation capacity in K14-IL-17A^ind/+^ aortas at all (Figure 3a,b). As described previously, vascular dysfunction in K14-IL-17A^ind/+^ mice was in line with an infiltration of Ly6G^+^/Ly6C^+^ neutrophil granulocytes into the aortic vessel wall [11,12]. The infiltration of Ly6G^+^/Ly6C^+^ neutrophils was not attenuated under telmisartan in K14-IL-17A^ind/+^ aortas (Figure 3c) in line with the persisting vascular dysfunction.

Taken together, high dose telmisartan treatment efficiently lowered systemic blood pressure in our murine model of severe psoriasis-like skin disease. However, it failed to reduce peripheral oxidative stress formation, vascular dysfunction, and inflammation correlating with the persistently inflamed skin.

## 3. Discussion

Our study points out for the first time that an effective antihypertensive treatment with telmisartan is possible in experimental psoriasis associated with hypertension and vascular disease. Also in humans, there is evidence for an increased cardiovascular risk of patients with severe psoriasis [2]. Yet the general awareness among physicians and patients is lacking. Reduction of the general cardiovascular risk profile in psoriasis patients is essential, including sufficient treatment of arterial hypertension [13]. Our IL-17A-driven mouse model of severe psoriasis combines the psoriasis-like skin inflammation with cardiovascular disease [11] as seen in patients with severe psoriasis. It is a convenient tool to study treatment options for improving concomitant cardiovascular disease in psoriasis. Considering the severe cutaneous and cardiovascular phenotype of this genetic model, the lowered systolic blood pressure indicates at least a partial benefit of ARB treatment. An efficient reduction of the classical cardiovascular risk factor hypertension in the psoriasis mice takes place, although the skin inflammation persists. However, one must be aware that vascular dysfunction and vascular inflammation were not attenuated at all, even though ARBs have been described to have anti-inflammatory properties [16,17]. The anti-inflammatory property of telmisartan might be not enough to rescue the phenotype of the chronic vascular inflammation in the K14-IL-17A^ind/+^ resulting from the severe skin inflammation and consequently systemic inflammation. However, we found an increased mortality in the group of ARB-treated psoriasis mice. This could indicate that the psoriatic mice do not tolerate changes in blood pressure as well as control mice, and this might be a consequence of the existing systemic inflammation. Blood pressure control and treatment in psoriasis seems to be more complicated than expected. Here, further studies are needed to clarify the exact mechanisms of arterial hypertension in psoriasis. Another open question is if ARB treatment reduces vascular inflammation in other, less severe, mouse models of psoriasis.

Vascular dysfunction/inflammation correlated with the persisting skin inflammation and the increased peripheral oxidative stress levels in psoriasis mice. It is most likely that the elevated blood pressure levels in psoriasis represent just the tip of the iceberg: While general antihypertensive treatment efficiently lowered the elevated systolic blood pressure, it did not attack the source of the cardiovascular disease associated with psoriasis, the persistent IL-17A-driven inflammation in the skin and vasculature. This finding is of crucial importance for the daily clinical practice. Efficient antihypertensive treatment in psoriasis is important, but it should not lead to a false sense of security that the cardiovascular comorbidity is thereby completely avoided.

Our findings contribute to the hypothesis that antihypertensive treatment might not be the leading factor for the prevention of cardiovascular disease and vascular inflammation in psoriasis. Just recently, studies from our group and others have linked the severity of psoriatic skin disease with the extent of vascular dysfunction and inflammation. We showed that successful anti-IL-17A treatment of psoriatic skin lesions diminished proinflammatory cytokines and vascular inflammation [12]. Furthermore, Dey et al. recently showed in a human cohort of psoriasis patients that improvement in skin disease was associated with improvement in aortic vascular inflammation [18]. Inhibitors of tumor necrosis factor in psoriasis showed a significant reduction in the risk of myocardial infarction compared with topical agents [19]. A Danish cohort study showed that systemic anti-inflammatory treatment with biological agents or methotrexate was also associated with lower cardiovascular disease event rates compared to patients treated with other anti-psoriatic therapies [20]. In light of these publications and our present findings, it is tempting to set up the possible equation of healthy skin equals healthy vessels. However, cardiovascular risk-factor reduction in the form of antihypertensive treatment does not reduce vascular inflammation as long as there is a persisting chronic IL-17A-driven inflammation affecting the whole body.

Translating the current literature into clinical practice, the successful treatment of cutaneous inflammation should not be the major goal just for dermatologists but should also reach the consciousness of cardiologists and general practitioners as it liberates the patients from chronic inflammation. In return, all involved parties need to be aware of the potentially increased cardiovascular risk of their, often young, psoriasis patients and should not miss the right and often early time point to initiate a therapeutic concept for all modifiable cardiovascular risk factors.

## 4. Materials and Methods

### 4.1. Mouse Models of Psoriasis-Like Skin Disease

IL-17A^ind/ind^ mice [21] were crossed to K14-Cre mice to obtain K14-IL-17A^ind/+^ [15,22]. We used IL-17-A^ind/+^ littermates as controls. Both mouse genders were used as we did not detect any differences between the genders in the analyses. K14-IL-17A^ind/+^ and IL-17A^ind/+^ control mice were treated with a high dose of telmisartan (40 mg/kg body weight/day) in drinking water for four weeks starting at the age of six weeks.

All mice were housed and treated in accordance with the laws and institutional guidelines of the Central Animal Facility of the University Medical Center Mainz, Germany. Experiments were approved by the Animal Care and Use Committee from the Land of Rhineland-Palatinate, approval number G 17-1-076 (29th of November, 2017).

### 4.2. Blood Pressure Measurements

Systolic blood pressure was obtained in mice using a tail-cuff, noninvasive blood pressure system with the CODA^®^ High Throughput System (Kent Scientific, Torrington, CT, USA). A minimum of five measurements was obtained from each mouse. The psoriasis-like skin affliction did not allow us to implant telemetric catheters as we usually do for blood pressure measurements, so we had to switch to non-invasive blood pressure measurements.

### 4.3. Psoriasis Area and Severity Index (PASI)

The severity of psoriasis-like skin disease was determined by modified PASI scoring, as described previously [11]. Briefly, erythema and scaling were scored (score range = 0–4), skin thickness of the ears and the back skin were measured using a caliper (μm), and the percentage of affected skin was determined. The cumulative PASI score was calculated for the K14-IL-17A^ind/+^ as follows: (erythema score + scaling score + skin thickness change [%]) × affected area [%].

### 4.4. Detection of ROS/RNS Formation with L-012-Enhanced Chemiluminescence

The oxidative burst of whole blood was determined by 8-amino-5-chloro-7-phenylpyridol[3,4-d]pyridazine-1,4-(2H,3H)dione sodium salt (L-012)-enhanced chemiluminescence. We injected 200 IU of heparin into the beating heart of the anesthetized mouse, and drew venous blood from the right ventricle. Enhanced chemiluminescence was counted in a volume of 200 μL per well of blood samples diluted to 1:50 in phosphate buffered saline containing Ca^2+^/Mg^2+^ (1 mmol/L) with L-012 (100 μmol/L) at intervals of 5 min using a Centro plate reader (Berthold Technology, Bad Wildbad, Germany). Enhanced chemiluminescence was expressed as counts per minute after incubation for 25 min.

### 4.5. Vascular Tone Experiments

The vascular responsiveness to vasodilators (increasing doses of ACh in a range from 10^−9^–10^−5.5^ mol/L and increasing doses of NTG in a range from 10^−9^–10^−4.5^) of isolated aortic rings was studied. Isolated aortas were cut into 4 mm segments and installed on force transducers (from Kent Scientific Corp., Torrington, CT, and from PowerLab, AD Instruments, Spechbach, Germany) in organ chambers filled with Krebs–Henseleit solution (98.93 mmol/L of NaCl, 4.69 mmol/L of KCl, 2.49 mmol/L of CaCl_2_, 1.2 mmol/L of MgSO_4_, 0.613 mmol/L of K_2_HPO_4_, 25 mmol/L of NaHCO_3_, 11.1 mmol/L of D-glucose, 37 °C, pH 7.35) bubbled with carbogen gas (95% O_2_/5% CO_2_). Indomethacin 10 μmol/L was added to prevent endogenous synthesis of prostaglandins. Aortic segments were stretched gradually over 30 min to reach a resting tension of 1.0 g. After the pre-constriction with phenylephrine (10^−8^–10^−5.5^ mol/L), cumulative concentration–relaxation curves were recorded in response to increasing concentrations of ACh or NTG.

### 4.6. Cytokine Detection

Cytokines were determined in heparinized plasma samples of mice. Levels of IL-17A, IL-6, granulocyte colony-stimulating factor (G-CSF), IL-1β, granulocyte-macrophage colony-stimulating factor (GM-CSF), interferon (INF)-γ, tumor necrosis factor alpha (TNF-α), IL-10, IL-12p40, IL-12p70, IL-13, IL-1α, IL-2, IL-4, IL-5, IL-9, chemokine (C-X-C motif) ligand (CXCL)1, chemokine (C-C motif) ligand (CCL)2, CCL3, and CCL4 were detected by using Bio-Plex Pro™ Mouse Cytokine Kit (Bio-Rad, Hercules, CA, USA) according to manufacturer’s protocol (Bio-Rad). The plate was analyzed by Luminex xMAP^®^ technology by the use of the MAGPIX Luminex Instrument (Thermo Fisher Scientific, Waltham, MA, USA).

### 4.7. Flow Cytometric Analysis

Aortas were digested using Liberase (1 mg/mL) for 30 min at 37 °C. Subsequently, cells were filtered through 70 μm cell strainers (Falcon, BD Biosciences) to obtain single-cell suspensions, which were pre-incubated in Fc-Block for at least 10 min. Cells were surface-stained with various combinations of fluorophore-conjugated antibodies at 4 °C for 30 min to discriminate B220^+^ B cells (clone: RA3-6B2), CD11b^+^ myeloid cells (clone: M1/70), Ly6C^+^Ly6G^+^ neutrophils (Ly6C, clone: AL-21; Ly6G, clone: 1A8), Ly6C^single+^ monocytes, and F4/80^+^ monocytes/macrophages (clone: BM8). Flow cytometric acquisition was performed on a FACS Canto II (BD Biosciences) and analyzed using FlowJo software (BD FlowJo, Ashland, OR, USA).

### 4.8. Statistical Analysis

Power calculation as a basis for any animal experiment according to the federal animal law was performed using G*Power Software Version 3.1.9.2. Effect sizes were calculated from previously published experiments. All findings shown have been reproduced in at least two independent experiments. Data are presented depending on their scale and distribution with the arithmetic mean and standard error of the mean (mean ± SEM). Outliers identified by ROUT or Grubbs’ test were excluded. Normality was assessed by the Kolmogorov–Smirnov normality test. To compare independent measurements, we used the two-tailed unpaired Student’s t-test and Mann–Whitney t-test, as appropriate. To compare more than two groups, we used one-way ANOVA followed by the Bonferroni’s post-hoc test or Kruskal–Wallis test followed by Dunn’s post hoc test, as appropriate. Statistical analysis was performed using GraphPad Prism 8 (version 8; GraphPad Software, Inc., La Jolla, CA, USA). The *p*-values of ≤0.001, ≤0.01, and ≤0.05 were considered statistically significant and marked by three, two, and one asterisks, respectively.

## Supplementary Materials

Supplementary materials can be found at https://www.mdpi.com/1422-0067/20/17/4261/s1.

## Abbreviations

ACE: Angiotensin-converting enzyme; AngII: Angiotensin II; ARB: Angiotensin II-receptor blocker; AT1: angiotensin II receptor type 1; BP: Blood pressure; IL: interleukin; G-CSF: Granulocyte-colony stimulating factor; PASI: Psoriasis Area and Severity Index; RAAS: renin-angiotensin-aldosterone system; ROS/RNS: reactive oxygen and nitric species; TNF-α: tumor necrosis factor alpha.
