# Supplementary material for: Telmisartan Lowers Elevated Blood Pressure in Psoriatic Mice without Attenuating Vascular Dysfunction and Inflammation
Source: Int J Mol Sci. 2019 Aug 30;20(17):4261. doi: 10.3390/ijms20174261 (PMC6747395; doi:10.3390/ijms20174261)
Supplement: Supplementary file 1 [file ijms-20-04261-s001.pdf]

Supplement

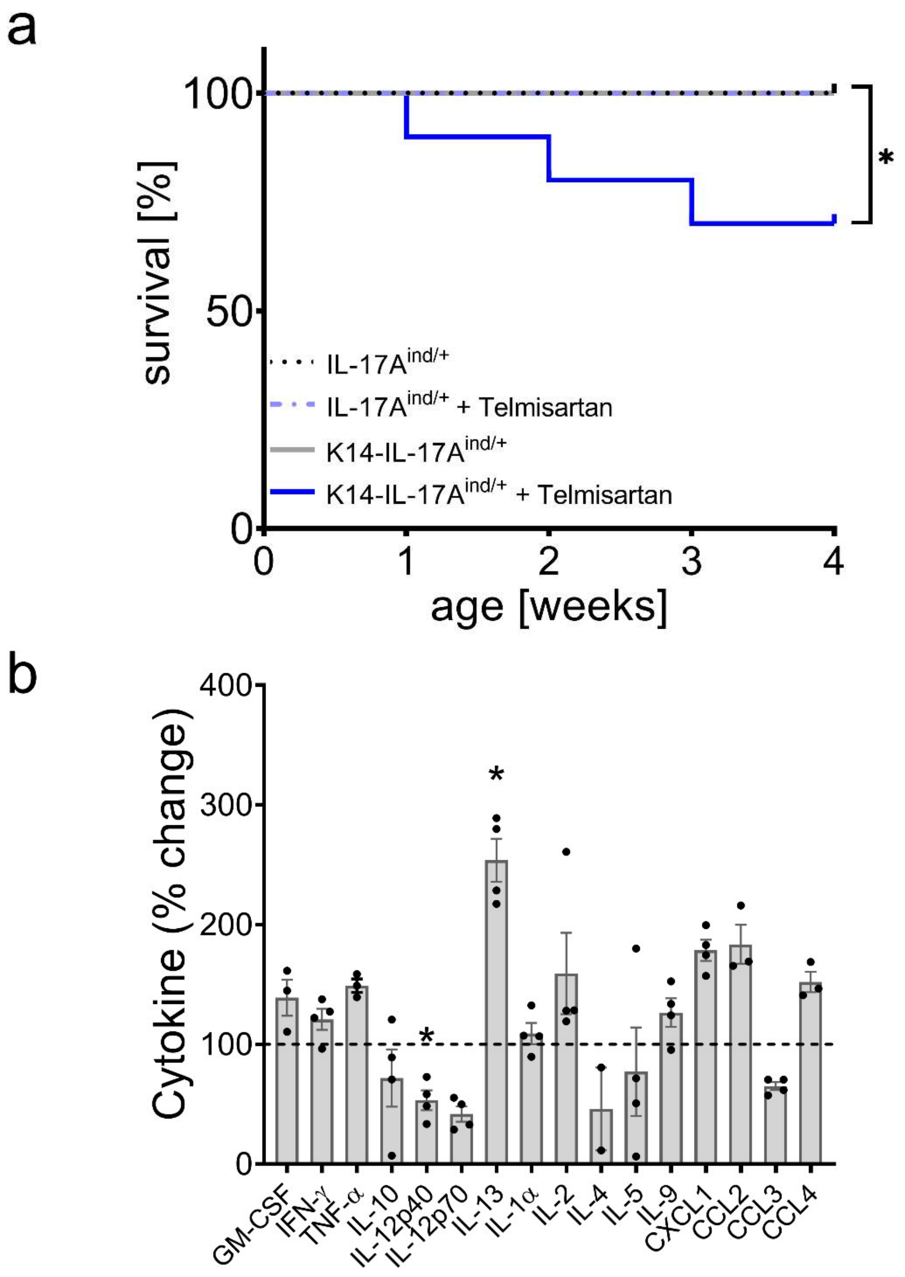

Supplementary Figure 1:

(a) Kaplan-Meier survival curve of K14-IL-17A$^{ind/+}$ and control mice with and without telmisartan treatment, n = 7-10 mice per group, Mantel-Cox test. (b) Cytokine profile in plasma of K14-IL-17A$^{ind/+}$ mice. Data was generated using the Bio-Plex assay kit in plasma of mice. IL-17A$^{ind/+}$ control mice were normalized to 100% (indicated with the dashed line), percentual change compared to control is shown for K14-IL-17A$^{ind/+}$ mice, n = 3-6, either Student's unpaired t-test or Mann-Whitney t-test. Data are presented as mean ± SEM.
